# Risk assessment and management of polypharmacy in the Geriatric Interdisciplinary Team: a case report

**DOI:** 10.3389/fmed.2025.1650502

**Published:** 2025-09-26

**Authors:** Jing-Wen Guo, He Zhao, Wen Tian, Shuang Cai

**Affiliations:** ^1^Department of Pharmacy, The First Affiliated Hospital of China Medical University, Shenyang, Liaoning, China; ^2^Department of Pharmacy, Affiliated Zhongshan Hospital of Dalian University, Dalian, Liaoning, China; ^3^Department of Geriatrics, The First Affiliated Hospital of China Medical University, Shenyang, Liaoning, China

**Keywords:** Geriatric Interdisciplinary Team, clinical pharmacists, polypharmacy, case report, individualized treatment

## Abstract

Older patients frequently experience the coexistence of multiple diseases. The conventional single-discipline diagnostic and treatment model is insufficient to comprehensively address all concurrent conditions, thereby necessitating a multi-disciplinary collaborative approach. The Geriatric Interdisciplinary Team represents an innovative diagnostic and treatment paradigm tailored for older patients, with clinical pharmacists serving as integral members of this team. This article presents a case report of the application of Geriatric Interdisciplinary Team, involving the participation of a clinical pharmacist, aiming to provide practical insights and experiences of this model for clinical pharmacists.

## 1 Introduction

With the accelerated aging process of the human population, changes in lifestyle, enhancements in socioeconomic conditions, and progressions in medical diagnostic technologies, the incidence rates of Multimorbidity have shown a significant upward trend. Globally, multiple chronic conditions and the resulting polypharmacy have become major public health challenges in aging societies. A systematic review and meta-analysis covering 54 countries revealed that over half of adult aged 60 and above suffer from multiple comorbidities (1). Moreover, long-term polypharmacy is significantly associated with a 30% increased risk of mortality and a 61% elevated risk of hospitalization (2). The traditional single-discipline treatment model cannot offer systematic diagnosis and treatment for this vulnerable population, so the Geriatric Interdisciplinary Team (GIT) has become the main treatment model for older patients in developed countries.

Currently, GIT in China is still in the initial trial stage and has only been used in a few general hospitals (3). In China, more than 50% of older patients concurrently suffer from three or more chronic diseases (4). Meanwhile, the rate of polypharmacy is as high as 70.8%, and the average daily intake of medications reaches 8.6 types (5). This frequently poses risks such as repeated medication usage, drug interactions, and heightened risks of adverse reactions, which in turn elevates medical costs and impacts clinical outcomes. To better address the health issues of olderly patients with comorbidities, our hospital has established the GIT. This article focuses on the perspective of clinical pharmacists’ participation in the practice of the geriatric multidisciplinary team, adopting a patient-centered approach. By optimizing medication regimens and implementing personalized medication monitoring, the safety of medication use is ensured, thereby minimizing drug interactions to the greatest extent.

**Figure 1:**
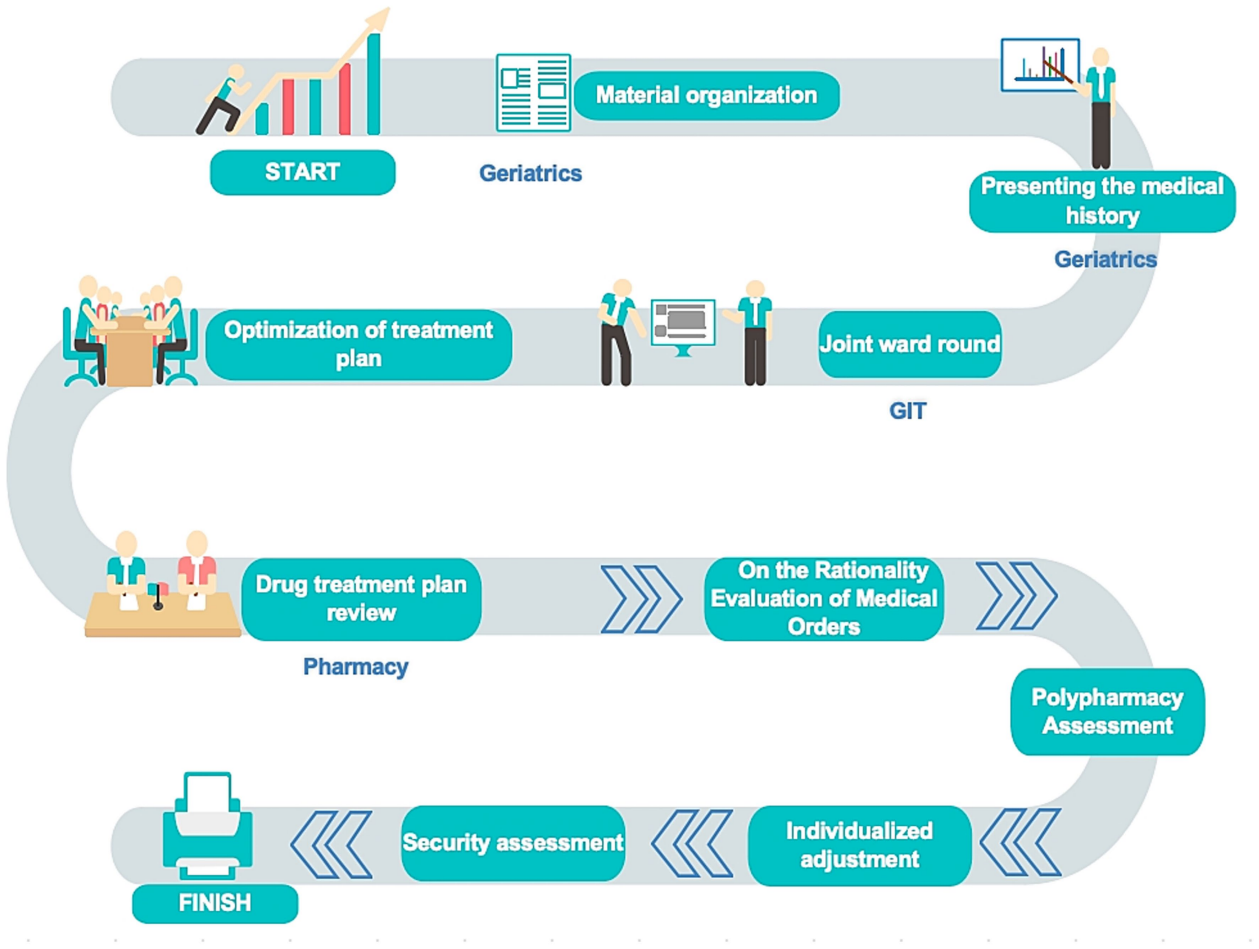
The workflow of GIT.

The GIT at our center is primarily composed of geriatricians, nurses, rehabilitation physicians, nutritionists, clinical pharmacists, and psychiatrists. The workflow proceeds as follows: Initially, geriatricians conduct comprehensive geriatric assessments to screen patients requiring GIT meetings. Subsequently, the team convenes where the geriatrician presents the medical history, followed by a joint bedside evaluation of the patient. The team then returns to the meeting room to discuss the patient’s clinical progress and optimize treatment plans. Clinical pharmacists are responsible for reporting pharmaceutical care records during hospitalization, identifying risk points in current medication regimens, and ultimately reviewing and consolidating medication-related risks from each specialty’s finalized treatment plans. Throughout this process, the team maintains effective information sharing and collaborative mechanisms (Figure 1).

## 2 Case description

A 79-year-old male patient, with a height of 172 cm and weight of 60 kg, had a 27-year history of coronary heart disease (CHD). He underwent coronary angiography and interventional therapy 27 years ago, 13 years ago, and 3 months ago, with a total of 3 stents implanted in the left anterior descending artery and left circumflex artery to date. He was on medication, with no recent recurrence of chest pain. Four days prior, he developed chest tightness, shortness of breath, and fatigue after climbing stairs, without chest pain or profuse sweating. Symptoms relieved after ~1 min of rest, and he was admitted to our hospital on March 1, 2025. He had no history of hypertension, but a 1-year history of arrhythmia with frequent premature ventricular contractions; 5-month history of mild-to-moderate aortic stenosis; and 7-month history of organizing pneumonia (on oral methylprednisolone). Elevated blood glucose was detected half a month ago, for which acarbose was prescribed. Past history also included atherosclerotic occlusive disease of bilateral anterior tibial arteries and osteopenia of bilateral hip joints.

On admission examination: crackles were auscultated in the lower lung fields; pulsation range was normal, heart rhythm regular, with no pathological murmurs in valve auscultation areas. Chest CT scan revealed ground-glass opacities in the left upper lung lobe, bilateral interstitial lung lesions, localized bilateral emphysema, and slightly enlarged mediastinal lymph nodes. Laboratory results: granulocyte count 7.15 × 10^9^/L, serum albumin 39.7 g/L, C-reactive protein 53.80 mg/L, procalcitonin 0.106 ng/mL, serum interleukin-6 (IL-6) 22.00 pg./mL; other tests were unremarkable.

Upon admission, medication reconciliation was performed for the patient. The patient’s medication orders and dosages remained consistent before and after admission, with no special adjustments made. We held a git meeting on March 5th and conducted a multidisciplinary joint ward round.

Admission diagnoses: organizing pneumonia with infection; stable CHD; post-coronary angiography and stent implantation; arrhythmia with frequent ventricular premature contractions; mild-to-moderate aortic stenosis; bilateral anterior tibial artery atherosclerotic occlusive disease; bilateral hip osteopenia.

Post-admission comprehensive assessment showed: mild dependence on activities of daily living; Geriatric Frailty Screening (FRAIL) Scale: 4 points, marked frailty; reduced calf circumference (left 34.5 cm, right 32.5 cm); Appendicular Skeletal Muscle Index (ASMI) 7.02; decreased 6-meter walk speed; abnormal time for five chair rises; normal grip strength; high fall risk; and at risk of malnutrition.

According to the American Geriatrics Society (AGS) (6) and the National Institute for Health and Care Excellence (NICE) (7), The guidelines for the diagnosis and treatment of comorbidities in older patients focus more on the patients treatment experience and preferences, aiming to improve disease symptoms and prevent complications from chronic diseases. Therefore, the key points discussed by the GIT team include: addressing the causes of shortness of breath; further optimizing nutritional and rehabilitation plans; actively intervening due to family reports of anxiety and pessimism; evaluating medication risks for patients taking multiple medications, simplifying prescriptions, and improving compliance.

Opinions of GIT members: ① Endocrinology Consultation Opinion: The patient presented with polydipsia, polyphagia, and polyuria, diagnosed with diabetes. Postprandial blood glucose elevated after taking methylprednisolone tablets. It was recommended to continue the current regimen of acarbose tablets (0.1 g chewed with lunch and dinner) for glycemic control, with regular blood glucose monitoring. If glycemic control was suboptimal, repaglinide tablets could be added for immediate oral administration before meals if necessary. Subsequently, the patient’s blood glucose was well-controlled without additional hypoglycemic agents. ② Rehabilitation Consultation Opinion: The patient was in the acute stage of the disease with unstable oxygen saturation; thus, rehabilitation exercises were not recommended temporarily. ③ Nutrition Consultation Opinion: The patient was advised to adhere to a low-carbohydrate, low-GI (glycemic index) diet with high-quality protein supplementation. During hospitalization, the patient’s weight remained stable, and serum albumin levels showed an upward trend. ④ Respiratory Consultation Opinion: Elevated blood glucose was detected half a month prior, prompting a reduction in methylprednisolone dosage from 12 mg/d to 8 mg/d. The current shortness of breath was attributed to intolerance to the reduced hormone dosage. It was advised to monitor oxygen saturation, adjust methylprednisolone back to 12 mg/d, and routinely recheck inflammatory markers. The patient’s shortness of breath improved thereafter. ⑤ Psychiatric Consultation Opinion: The patient was attentive to their condition and, due to personality traits, exhibited mild illness anxiety. No medication intervention was needed temporarily; family members were advised to provide necessary support and encouragement to help the patient maintain a positive attitude. ⑥ Clinical Pharmacist Intervention: Details of the patient’s medications and interventions are presented in Table 1.

**Table 1:** Detailed information of patients medication and intervention.

Purpose of medication	Name of drug	Dosage and administration	Frequency of administration	Adjust recommendations	Adjust the results
Liver protection	Glutathione tablets	0.4 g	tid	Block up	Discontinue glutathione tablets and continue to use polyene phosphatidylcholine capsules
	Polyene phosphatidylcholine capsules	456 mg	tid		
Organized pneumonia	Methylprednisolone tablets	12 mg	qd	Monitor blood sugar and be alert for gastrointestinal ulcers and osteoporosis	The patients’ blood sugar was stable, and no new adverse reactions were reported
Stable coronary heart disease	Isosorbide mononitrate sustained release tablets	40 mg	qd	Continue to apply	Continue to apply
	Metoprolol succinate sustained release tablets	23.75 mg	qd	There are many interactions and it is recommended to switch to another drug	Switch to Bisoprolol fumarate tablets
Arrhythmia	Propafenone hydrochloride tablets	150 mg	tid	Continue to apply	Continue to apply
	Diltiazem sustained release capsules (II)	90 mg	qd		
Antiplatelet	Ticagrelor tablets	60 mg	bid	Continue to apply	Continue to apply
Reduce blood lipids, stabilize plaques	Rosuvastatin calcium tablets	10 mg	qd	Monitor creatine kinase and transaminase	Creatine kinase and transaminase were normal
	Ezetimibe tablets	10 mg	qd		
Lower blood sugar	Acarbose tablets	100 mg	bid	Continue to apply and monitor blood sugar	The patients’ blood sugar was stable
Anti-infection	Moxifloxacin hydrochloride injection	0.4 g	qd	Monitor blood sugar and electrocardiogram	The patients’ blood sugar was stable and the electrocardiogram was normal
Supplement nutrients	Compound amino acid capsules	1 pill	bid	Continue application with a break from moxifloxacin	Continue application with a break from moxifloxacin
	Multivitamin tablets	1 tablet	qd		
Protect the stomach	Rebamipide Tablets	100 mg	tid	Continue to apply	Continue to apply
	Pantoprazole Enteric-Coated Tablets	40 mg	qd	Discontinue medication after regular course of treatment	No new stomach discomfort
Bilateral hip osteopenia	Vitamin D3 Soft Capsules	1,000 IU	qd	Monitor the level of 25-hydroxyvitamin D	The 25-hydroxyvitamin D level is normal

## 3 Discussion

On the Rationality Evaluation of Medical Orders: The clinical pharmacist meticulously reviews all medications utilized by the patient post-admission, assessing their appropriateness, dosage, contraindications, and potential for drug interactions. Based on the patient’s admission test results, which indicated normal liver function, the administration of glutathione tablets and polyene phosphatidylcholine capsules is deemed off-label. For the management of chronic gastritis, the patient was prescribed pantoprazole enteric-coated tablets, also an off-label usage. It is advisable to discontinue these medications following standardized treatment protocols.

Polypharmacy Assessment: The patient exhibits multiple comorbidities and encounters challenges related to polypharmacy, which heightens concerns regarding the potential for inappropriate medication use (referred to as potentially inappropriate medication, PIM) among older patients due to the concurrent use of numerous medications. Clinical pharmacists conducted an evaluation of PIMs in older patients by employing the 2023 American Geriatrics Society (AGS) Beers Criteria (8), the Chinese older patients Potential Inappropriate Medication Use Judgment Standard (9), and relevant drug labels. PIM assessment results: (1) PIMs to be Avoided in the older patients: Proton pump inhibitors (PPIs) elevate the risk of *Clostridium difficile* infection, pneumonia, gastrointestinal malignancies, bone loss, and fractures in older patients, and should not be administered for more than 8 weeks. Research indicates that the concurrent use of oral corticosteroids and PPIs heightens the risk of osteoporotic fractures by 1.6 times (10). In this case, the patient has a history of PPI use spanning 1 year and is currently taking glucocorticoids, thereby increasing the risk of fractures; it is advisable to discontinue PPIs. (2) Medications that should be used with caution in the older: as ticagrelor elevates the risk of major bleeding in older adults, particularly those aged 75 years or older. An analysis from a Chinese cardiovascular disease quality improvement project study revealed that among patients aged 75 years or older with acute coronary syndrome who underwent ticagrelor treatment, the risk of significant intra-hospital bleeding rose by 45% (11). Given that the patient is 79 years old, ticagrelor should be used with utmost caution. (3) Potential drug interactions to be avoided in older patients: The combination of rosuvastatin and ezetimibe can increase the risk of muscle damage and elevate transaminase levels. When diltiazem is used in combination with ticagrelor, ticagrelor is mainly metabolized by CYP3A4, while diltiazem is an inhibitor of CYP3A4. Therefore, the combination of the two will increase the blood concentration of ticagrelor, leading to an increase in the risk of bleeding. When diltiazem is used in combination with metoprolol, diltiazem is a calcium channel blocker. When used in combination with beta-blockers, it has an additive inhibitory effect on atrioventricular conduction and sinoatrial function. There have been case reports of significant bradycardia when beta-blockers are used in combination with calcium channel blockers (12, 13); Propranolol and metoprolol used together, where metoprolol is metabolized via CYP2D6, and propranolol acts as an CYP2D6 inhibitor, can increase the blood concentration of metoprolol by 2 to 5 times. It also has *β* receptor blocking effects; it is recommended to switch to bisoprolol to reduce interactions; Diltiazem and methylprednisolone used together, where methylprednisolone is primarily metabolized via CYP3A4, and diltiazem is an inhibitor of CYP3A4, combined use can increase the risk of cardiac suppression; In non-diabetic individuals, the incidence of hyperglycemia after receiving medium to high doses of glucocorticoids exceeds 28–45% (14); In addition, patients who used both hypoglycemic drugs (acarbose) and moxifloxacin were reported to have impaired blood glucose, especially prone to hypoglycemia (15); The combination of mosaisacin and metoprolol increases the effect of prolongation of QT interval. There have been many reports of QT interval prolongation caused by mosaisacin, and it causes torsades de pointes in high-risk patients (16); The combination of moxifloxacin with multivitamins can significantly affect its absorption, leading to lower than expected blood levels of moxifloxacin. There are no inappropriate drugs with drug-disease or drug-syndrome interactions; there are no drugs that should be avoided or reduced in dose due to decreased renal function in the older.

Individualized adjustment: Based on the patients preference, the liver protection drug was continued in the absence of abnormal liver function. After communication and discussion with the psychiatrist, the patient finally agreed to stop using glutathione tablets and continue using polyene phosphatidylcholine capsules. The patients liver and kidney function were normal and there were no contraindications for related drugs.

### 3.1 Security assessment

No drug-related discomfort was observed during hospitalization.

### 3.2 Intervention outcomes

Throughout the intervention, patient-centered, multidisciplinary collaboration enabled individualized diagnosis and treatment for the elderly patient. Clinical pharmacists performed prescription reconciliation on physicians’ orders and assisted in monitoring drug-related adverse reactions and interactions, ensuring the safety and efficacy of medication use. Following intervention by our hospital’s geriatric multidisciplinary integrated team, no adverse events such as falls occurred during hospitalization. The patient had stable blood glucose and blood pressure, improved shortness of breath, a 1 kg weight gain, and enhanced nutritional status. At 1-month follow-up, the patient maintained stable blood pressure and glucose, normal liver and kidney function, and a normal serum albumin level. Anxiety symptoms improved, medication adherence was enhanced, and family support was high.

While GIT care has become an established model in some Western countries, it has not been widely adopted in China. This limited implementation arises from challenges within China’s resource-constrained healthcare system: First, the lack of long-term multicenter studies on GIT in China leaves the sustained benefits for older adults unclear. Second, overburdened medical teams, facing growing workloads, must selectively enroll patients in GIT. This project has adapted the GIT model through localization strategies, featuring a standing multidisciplinary expert team that holds biweekly GIT meetings. Prior to these meetings, clinical teams share patient materials with expert groups, allowing specialists to review cases and anticipated treatment goals in advance. A key innovation is the integration of embedded clinical pharmacists. Within GIT, pharmacists act not only as “medication experts” but also as standardization drivers: they establish standards for core processes such as medication assessment, regimen optimization, and adverse reaction monitoring, providing a basis for formulating national guidelines. Through rational medication interventions, they reduce medical waste, demonstrating the cost-effectiveness of GIT medication management and enhancing hospital efficiency under the Diagnosis Related Groups (DRG)/Diagnosis-Intervention Packet (DIP) payment systems and pharmaceutical zero-markup reforms. Additionally, pharmacists optimize physicians’ treatment plans by addressing drug interactions, dosage form suitability, and adherence. Currently, most domestic and international GIT case reports are physician-led, whereas this case, from the perspective of clinical pharmacists, focuses on medication adjustment and full-course patient monitoring. Through the full participation of clinical pharmacists in multidisciplinary ward rounds, medication optimization efficiency has been improved. This further underscores the unique value of this study in terms of localized practice, the deepened role of pharmacists, and cost-effectiveness.

### 3.3 Novelty

This study provides new insights into how pharmacist-led interventions enhance medication safety, improve cost efficiency, and optimize clinical outcomes in geriatric patients. The study not only established a pharmaceutical hierarchical care standard tailored to the characteristics of older patients, but also incorporated evidence-based references including the 2023 Beers Criteria and the Chinese criteria for potentially inappropriate medications in older adults.

### 3.4 Scientific significance

Our findings contribute to the growing body of evidence supporting pharmacists’ role in polypharmacy management, to provide standardized protocols for pharmacists in implementing polypharmacy management in GIT, with potential implications for clinical practice and healthcare policy.

## Data Availability

The raw data supporting the conclusions of this article will be made available by the authors, without undue reservation.
